# Multimodal imaging features of non-proliferative and proliferative diabetic retinopathy based on SD-OCT and fundus autofluorescence

**DOI:** 10.3389/fmed.2026.1779259

**Published:** 2026-03-02

**Authors:** Zixun Wang, Chenxi Ji, Xiaoxue Hu, Tingyu Zhang, Tingxi Liu, Xiaoling Zhang, Yuhang Wang, Zhiqing Li, Xiuhong Qin, Ming Sun

**Affiliations:** 1 Tianjin Key Laboratory of Retinal Functions and Diseases, Tianjin Branch of National Clinical Research Center for Ocular Disease, Eye Institute and School of Optometry, Tianjin Medical University Eye Hospital, Tianjin, China; 2 The First Affiliated Hospital of Dalian Medical University, Dalian, Liaoning, China; 3 Xi’an Gaoling District Hospital, Xi’an, Shaanxi, China; 4 Wuhan Children’s Hospital (Wuhan Maternal and Child Healthcare Hospital), Tongji Medical College, Huazhong University of Science & Technology, Wuhan, Hubei, China; 5 Handan Eye Hospital (The Third Hospital of Handan), Hebei, China

**Keywords:** diabetic retinopathy, fundus autofluorescence, imaging biomarkers, macular edema, optical coherence tomography

## Abstract

**Purpose:**

To characterize and compare multimodal imaging features of non-proliferative diabetic retinopathy (NPDR) and proliferative diabetic retinopathy (PDR) using spectral-domain optical coherence tomography (SD-OCT) and fundus autofluorescence (FAF).

**Methods:**

This cross-sectional observational study included 132 patients (219 eyes) with DR (120 NPDR eyes and 99 PDR eyes) and 73 healthy controls (129 eyes). All participants underwent comprehensive ophthalmic examinations, including fundus photography, fundus fluorescein angiography (FFA), FAF, and SD-OCT. OCT biomarkers, including hyperreflective foci (HRF), intraretinal cystic cavities (IRC), diabetic macular edema (DME), disorganization of retinal inner layers (DRIL), epiretinal membrane (ERM), posterior vitreous detachment (PVD), subretinal fluid (SRF), and disruption of the external limiting membrane (ELM) and ellipsoid zone (EZ) were systematically evaluated and compared between groups.

**Results:**

Hyperreflective foci, IRC, DME, PVD, and ERM were significantly more frequent in PDR eyes than in NPDR eyes (all *P* < 0.05). ERM was highly prevalent in both NPDR (80.0%) and PDR (84.8%) eyes. FAF effectively demonstrated intraretinal and preretinal hemorrhages, DME, and fibroproliferative membranes, while SD-OCT provided superior visualization of microstructural retinal alterations. Subfoveal choroidal thickness was significantly increased in both NPDR and PDR compared with healthy controls (*P* < 0.05), but did not differ considerably between NPDR and PDR.

**Conclusion:**

Spectral-domain optical coherence tomography and FAF provide complementary information for evaluating structural and functional retinal alterations in DR. FAF is particularly useful for visualizing hemorrhage, DME, and fibroproliferative membranes. In contrast, SD-OCT enables detailed assessment of multiple retinal biomarkers. The high prevalence of epiretinal membranes highlights their potential role in DR progression.

## Introduction

Diabetic retinopathy (DR) is one of the most common and serious microvascular complications of diabetes and is one of the leading causes of blindness in China (1). According to statistics, the number of people with visual impairment or blindness due to DR has increased significantly, and the prevalence has continued to rise from 1990 to 2020 (2). It is widely recognized that the risk factors for DR include diabetes duration, glycated hemoglobin (HbA1c) levels, hypertension, pregnancy, hyperlipidemia, nephropathy, and alcohol abuse (3). The study by Perais et al. demonstrated that, regardless of the severity of DR, the degree of good glycemic control helps reduce the risk of progression to proliferative diabetic retinopathy (PDR) (4). In the early stages of DR, patients are often asymptomatic. However, as the disease progresses, serious complications such as vitreous hemorrhage and tractional retinal detachment may occur, ultimately leading to blindness.

Fundus fluorescein angiography (FFA) remains the gold standard for diagnosing DR, with clear advantages for identifying microvascular abnormalities and retinal neovascularization. However, as FFA is an invasive procedure with contraindications, its application is limited. Clinically, spectral-domain optical coherence tomography (SD-OCT) for detecting biological imaging biomarkers holds broad application prospects. As early as 1988, Hee et al. found that OCT aids in diagnosing early DR microlesions, including macular retinal thickening (5). An increasing number of studies suggest that OCT-based biological imaging biomarkers can serve as new indicators of visual prognosis and treatment strategies in DR patients. These indicators include intraretinal cysts (IRC), disorganization of inner retinal layers (DRIL), hyperreflective foci (HRF) in the outer retina, and the integrity of the ellipsoid zone (EZ) and external limiting membrane (ELM), as well as diabetic macular edema (DME).

In addition, autofluorescence primarily detects retinal lipofuscin, which reflects the function of the retinal pigment epithelium (RPE). FAF is most used in age-related macular degeneration (AMD). Fleckenstein et al. summarized various early manifestations of AMD, including multifocal and mottled hyperautofluorescent dots (6). In advanced AMD, FAF can reveal hypoautofluorescent geographic atrophy lesions. Currently, there is limited research on FAF in DR, but studies have shown that petal-like macular edema can be clearly visualized on FAF.

The objective of this study was to describe the features of biological imaging biomarkers in patients with non-proliferative diabetic retinopathy (NPDR) and proliferative diabetic retinopathy (PDR) using OCT and FAF.

## Methods

### Patients and methods

This study was approved by the ethics committee of the First Affiliated Hospital of Dalian Medical University and Tianjin Medical University Eye Hospital [No: PJ-KS-KY-2023-288; PJ-KS-KY-2025-22]. All study procedures adhered to the principles outlined in the Declaration of Helsinki. All procedures involving comprehensive physical examinations and ophthalmic assessments were performed with participants’ informed consent.

We selected 132 consecutive patients (219 eyes) with DR. The NPDR group comprised 69 patients (120 eyes), the PDR group comprised 63 patients (99 eyes), and the health control group comprised 73 participants (129 eyes). The diagnosis of diabetic retinopathy was based on the following criteria: (1) history of diabetes mellitus; (2) the fundus manifestations of diabetic retinopathy include microaneurysms, hemorrhages, exudates, cotton-wool spots, neovascularization, and proliferative membranes; and (3) absence of retinopathy secondary to other similar diseases, such as retinal vein occlusion, retinal periphlebitis, retinal artery occlusion, hypertensive retinopathy or macular diseases; (4) absence of those who were allergic to sodium fluorescein; (5) absence of those of refractive media opacity affecting image quality; (6) exclude patients with pathological myopia and an axial length (AL) > 26 mm.

All patients were given an ophthalmologic examination, including intraocular pressure, slit lamp examination, scanning Laser Ophthalmoscopy (Daytona, P200T), fundus fluorescein angiography, and fundus autofluorescence (Heidelberg Engineering, Heidelberg, Germany). Optical coherence tomography scans were performed using a Spectralis (Heidelberg Engineering, Heidelberg, Germany). Sections were obtained from line scans across several important lesions with 100 average B-scans per image, as well as a dense volume scan encompassing the whole lesion with 30 average B-scans per image (8.6 mm × 8.6 mm rectangular area). Considering the intrinsic dynamic changes in choroidal thickness, all examinations were conducted daily between 9:00 AM and 11:00 AM, and clear images of the subfoveal choroid were acquired using the enhanced depth imaging (EDI) mode of the Heidelberg Spectralis OCT (Heidelberg Engineering, Germany). Subfoveal choroidal thickness (SFCT) was measured 3 times using the optical coherence tomography system software by one observer. The final data were calculated as the average of three measurements. The SFCTs were compared using the Chi-square test. To account for age differences between groups, a multivariate linear regression was performed to evaluate the independent effect of DR status on SFCT, controlling for age. Categorical OCT features were summarized as counts and percentages. Comparisons between NPDR and PDR eyes were performed using the chi-square test, or Fisher’s exact test when expected cell counts were <5. All statistical analyses were conducted using SPSS version 23.0, with statistical significance set at *P* < 0.05. The *p*-value for age was calculated using a one-way ANOVA, representing the baseline demographic difference between healthy controls and DR patients. Multivariate analysis was employed to adjust for age-related SFCT.

## Results

Of our 132 patients (219 eyes), 78 (59.09%) were male, and 54 (40.91%) were female. Patient ages ranged from 23 to 76 years (mean age 52.75 ± 14.17 years). Among these 132 patients, 120 (54.79%) eyes had NPDR, and 99 (45.21%) had PDR. Seventy-three healthy individuals with normal characteristics were selected as the control group, with an average age of 37.33 ± 15.72 years. Basic information on this study is shown in Table 1, and the optical coherence tomography features of NPDR and PDR are summarized in Table 2. Compared with NPDR, eyes with PDR showed a significantly higher prevalence of hard exudates (69.7% vs. 42.5%, *p* < 0.001), cotton-wool spots (48.5% vs. 22.5%, *p* < 0.001), and hyperreflective foci (87.9% vs. 62.5%, *p* < 0.001). Posterior vitreous detachment was also more frequently observed in PDR eyes than in NPDR eyes (72.7% vs. 45.0%, *p* < 0.001). Intraretinal cystic cavities and macular edema were significantly more common in PDR eyes (48.5% vs. 32.5%, *p* = 0.023; and 93.9% vs. 40.0%, *p* < 0.001), indicating more severe retinal structural involvement in advanced disease stages. In contrast, no significant differences were found between the two groups regarding subretinal fluid, disorganization of retinal inner layers, epiretinal membrane, or disruption of the external limiting membrane and ellipsoid zone (all *p* > 0.05). Notably, preretinal hemorrhage and fibrous proliferative membrane were observed exclusively in the PDR group (9.1% and 27.3%, *p* < 0.01), reflecting hallmark features of proliferative disease.

**TABLE 1:** Basic information form.

Characteristics	Healthy control (*n* = 73)	NPDR (*n* = 69)	PDR (*n* = 63)	*P*-value
Eyes (*n*)	129	120	99	–
Age (years)	37.33 ± 15.72	51.80 ± 14.20	53.80 ± 14.10	<0.001^a^
Gender (M/F)	42/31	41/28	37/26	0.854^b^

*^a^*Calculated by ANOVA.

*^b^*Calculated by Chi-square test.

**TABLE 2:** Optical coherence tomography (OCT) features in diabetic retinopathy (DR).

OCT features	NPDR (*n* = 120)	PDR (*n* = 99)	*P*-value
Microaneurysm	120	99	/
Intra retinal hemorrhage	120	99	/
Hard exudates	51	69	*P* < 0.001
Cotton wool spot	27	12	*P* < 0.001
Hyperreflective foci (HRF)	75	87	*P* < 0.001
Subretinal fluid (SRF)	15	6	*P* = 0.168
Disorganization of retinal inner layers (DRIL)	27	27	*P* = 0.510
Disruption of external limiting membrane and ellipsoid zone	18	6	*P* = 0.059
Epiretinal membrane (ERM)	96	84	*P* = 0.450
Posterior vitreous detachment (PVD)	54	72	*P* < 0.001
Intra retinal cystic cavity (IRC)	39	48	*P* = 0.023
Macular edema	48	93	*P* < 0.001
Preretinal hemorrhage	0	9	*P* = 0.002
Fibrous proliferative membrane	0	27	*P* < 0.001

### The manifestations of DR in SD-OCT, FAF, and FFA examination

#### The microaneurysm

**FIGURE 1:**
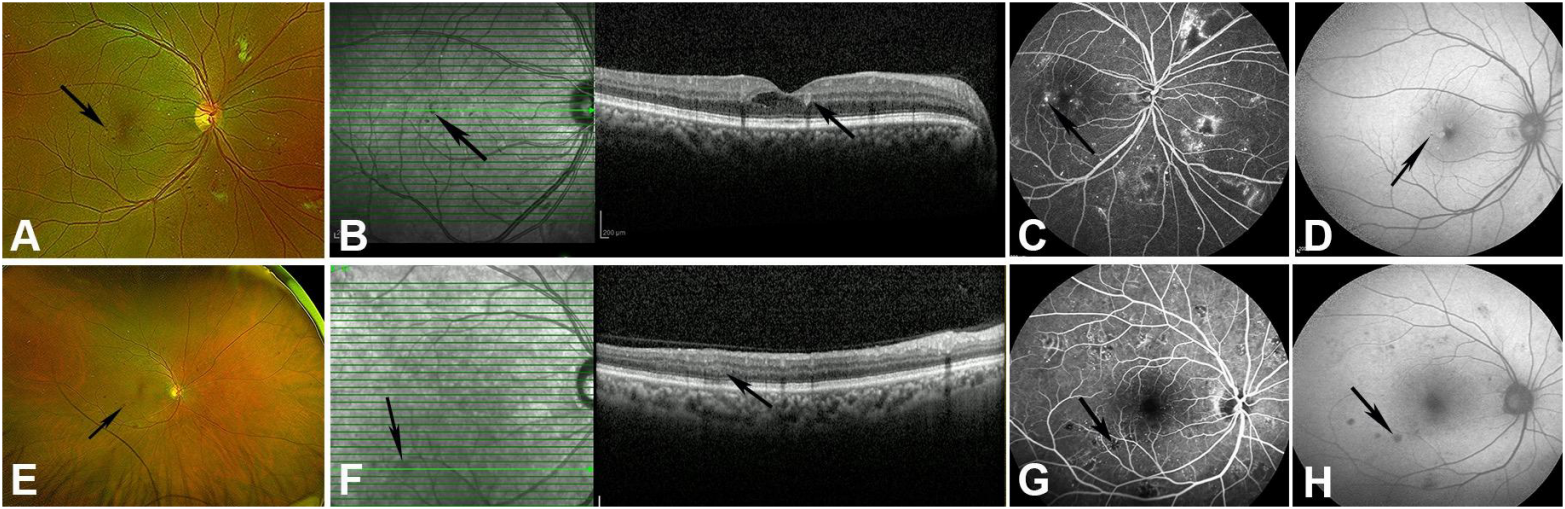
The manifestations of MA and intra-retinal hemorrhage in NPDR patients are observed in fundus photography, SD-OCT, FFA, and FAF examination. **(A)** Typical MA (black arrow) on fundus photography. **(B)** MA manifested as moderately reflective punctate lesions located in the inner retinal layers, accompanied by underlying artifacts on SD-OCT (black arrow). **(C)** FFA shows microaneurysms as punctate hyperfluorescence at the same location (black arrow). **(D)** Weak fluorescence changes of MA on FAF (black arrow). **(E)** Intra-retinal hemorrhage (black arrow) on fundus photography. **(F)** Hyperreflective lesions with accompanying artifacts (black arrow) of intraretinal hemorrhage on SD-OCT. **(G)** Blocked fluorescence of retinal hemorrhage on FFA (black arrow). **(H)** Blocked fluorescence of the same hemorrhage on FAF (black arrow).

Scanning Laser Ophthalmoscopy (SLO) revealed microaneurysms in all patients with NPDR and PDR (Figure 1A). FFA reveals microaneurysms and punctate hyperfluorescence in any part of the retina, which may leak fluorescence at a late stage (Figure 1C). The characteristic OCT findings of microaneurysms are moderately reflective, punctuate lesions larger than 30 μm in diameter located in the inner retinal layers, and may be accompanied by underlying artifacts (Figure 1B). Microaneurysms are not clearly visible on FAF; only larger, typical microaneurysms are visible, showing weak fluorescence changes consistent with their size (Figure 1D). In some patients with PDR, changes such as neovascularization and large areas of non-perfusion (NP) are observed, and the number and extent of microaneurysms are fewer than in NPDR on FFA examination.

#### Intra-retinal hemorrhage

The SLO examination revealed scattered or numerous hemorrhages on the retina, which can be classified as deep hemorrhage (Figure 1E) featuring punctate red dots or slightly larger round hemorrhage, and superficial hemorrhage appeared as small patchy or linear hemorrhage. In the 120 eyes with NPDR patients, all affected eyes (100%) showed intraretinal hemorrhage. FFA shows retinal hemorrhage with blocked fluorescence, varying in size with clear boundaries (Figure 1G). The SD-OCT scans revealed that all affected eyes (100%) of NPDR patients exhibited intraretinal hemorrhages, manifested as hyperreflective lesions located in the inner retinal layers or on the retinal surface, varying in size and quantity, with accompanying artifacts beneath them (Figure 1F). FAF shows intra-retinal hemorrhage presenting as uniformly sized hypofluorescent changes (blocked fluorescence) that can obscure the autofluorescence of the RPE (Figure 1H).

In PDR patients, all 99 affected eyes (100%) presented obvious intraretinal hemorrhage on fundus photography, including both deep and superficial hemorrhages. Compared to NPDR patients, PDR patients have more extensive and numerous intraretinal hemorrhages on the retina. FFA shows intraretinal hemorrhage presenting as uniformly sized, blocked fluorescence, which can occur in non-perfusion areas. Similarly, the blocked fluorescence of the retinal hemorrhage can be observed in all PDR-affected eyes on FAF examination. Also, intra-retinal hemorrhage can be found in all affected eyes (100%) by OCT examination.

#### Hard exudates

**FIGURE 2:**
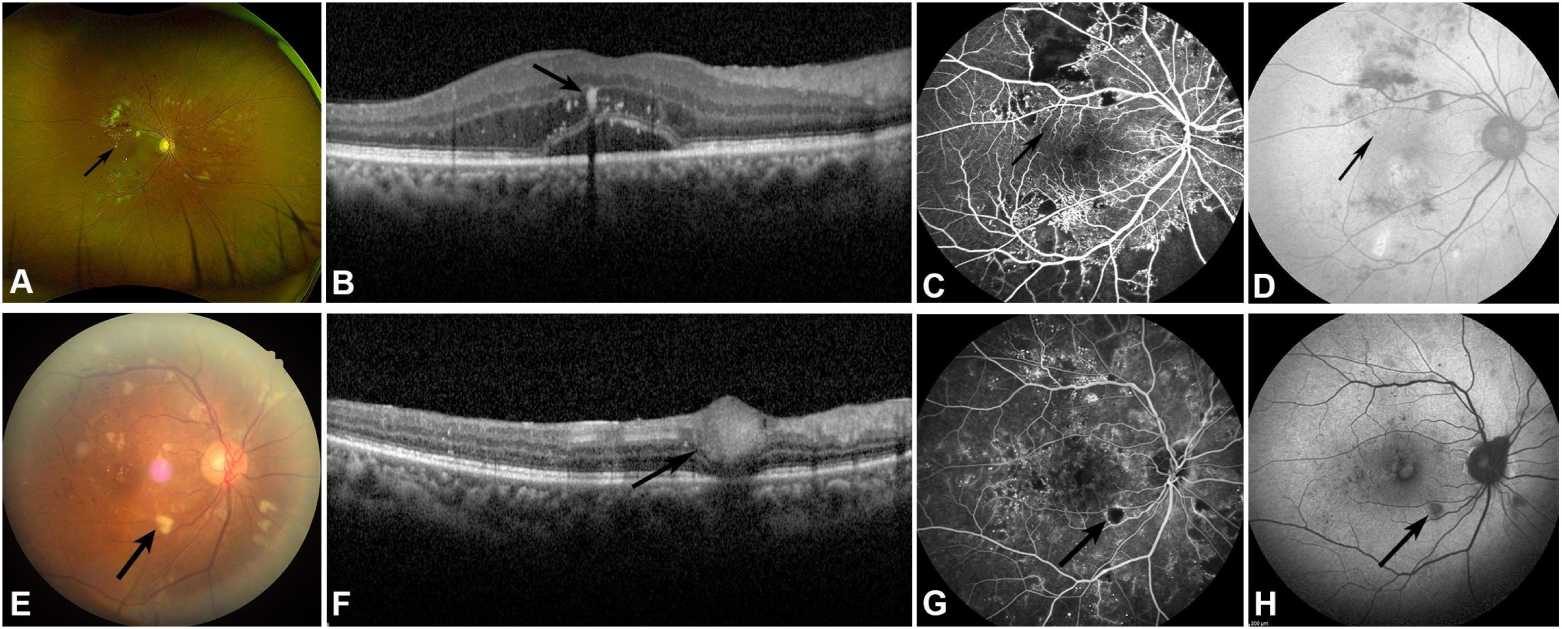
The manifestations of hard exudates and cotton wool spots in NPDR patients in fundus photography, SD-OCT, FFA, and FAF examination. **(A)** Typical hard exudate (black arrow) on fundus photography. **(B)** Hard exudate manifested as punctate hyperreflective materials in the outer plexiform layer of the retina, accompanied by posterior shadowing on SD-OCT (black arrow). **(C)** FFA shows hard exudate as hypofluorescent foci (black arrow). **(D)** Weak fluorescence changes of hard exudate on FAF (black arrow). **(E)** Cotton wool spot (black arrow) on fundus photography. **(F)** Thickened retinal nerve fiber layer lesions with high reflectivity (black arrow) of cotton wool spot on SD-OCT. **(G)** Hypofluorescence foci with NP areas underneath the cotton wool spot on FFA (black arrow). **(H)** Hypofluorescence foci of the same cotton wool spot on FAF (black arrow).

Among the 120 eyes of NPDR patients, 60 eyes (50%) showed hard exudates in fundus photography, featured as yellowish-white patchy spots of varying sizes with clear boundaries (Figure 2A), predominantly located in the macular but also scattered in the peripheral retina. The SD-OCT examination of the macular region revealed that among 120 eyes of patients with NPDR, 51 eyes (42.5%) exhibited typical hard exudates. Hard exudates on SD-OCT are characterized by multiple or clustered punctate hyperreflective materials in the outer plexiform layer of the retina, with a diameter greater than 30 μm, accompanied by posterior shadowing (Figure 2B). Since OCT examination was only performed in the macular area of the retina, hard exudates in these 51 eyes were all located in the posterior pole of the retina. Compared with fundus photography, OCT identified nine additional eyes with hard exudates in the peripheral retina that were not visible on fundus photography. FFA shows hypofluorescent foci consistent with hard exudates in fundus photography, with a weaker blocking effect than hemorrhage and no fluorescent leakage (Figure 2C). Hard exudates appear as hypofluorescent foci on FAF that correspond in size to those seen on fundus photography, with fluorescence intensity also lower than that of hemorrhage (Figure 2D). Among 120 affected eyes of patients with NPDR, only 30 eyes can exhibit hypofluorescent foci of hard exudates.

Compared with NPDR patients, hard exudates were observed in 69 of 99 PDR eyes (69.7%) on fundus photography and were found in the macula in all 69 eyes. The SD-OCT revealed punctate hyperreflective lesions in the outer plexiform layer of the macula in 69 eyes of PDR patients, consistent with the number of cases observed in fundus photography. FFA shows that 69 eyes of PDR patients had observable hypofluorescent foci of hard exudates. However, among the 99 affected eyes of PDR patients, only 27 eyes exhibited hypofluorescent foci corresponding to hard exudates, indicating that FAF has limited resolution for detecting hard exudates.

#### Cotton-wool spot

Fundus photography revealed cotton-wool spots in 36 of 120 eyes (30%) of NPDR patients, manifesting as irregularly shaped, ill-defined, variably sized, yellowish-white, cotton-wool-like lesions scattered on the retina (Figure 2E), predominantly in the posterior pole but also found in the peripheral retina. SD-OCT examination results showed that among 120 eyes of patients with NPDR, cotton wool spots were detected in 27 eyes (22.5%). The OCT features of cotton wool spots manifest as thickened retinal nerve fiber layer lesions with high reflectivity, accompanied by inner retinal edema and obscuration of posterior structures (Figure 2F). Compared with fundus photography, there were 12 additional eyes with cotton wool spots located in the peripheral retina that were not visible on OCT, because the OCT examination was only performed in the macular area of the retina. FFA shows cotton-wool spots as island-like or patchy hypofluorescent foci, with NP areas beneath and around them (Figure 1G). FAF indicates cotton wool spots as hypofluorescent foci, with weaker fluorescence blockage compared to hemorrhage (Figure 1H).

**FIGURE 3:**
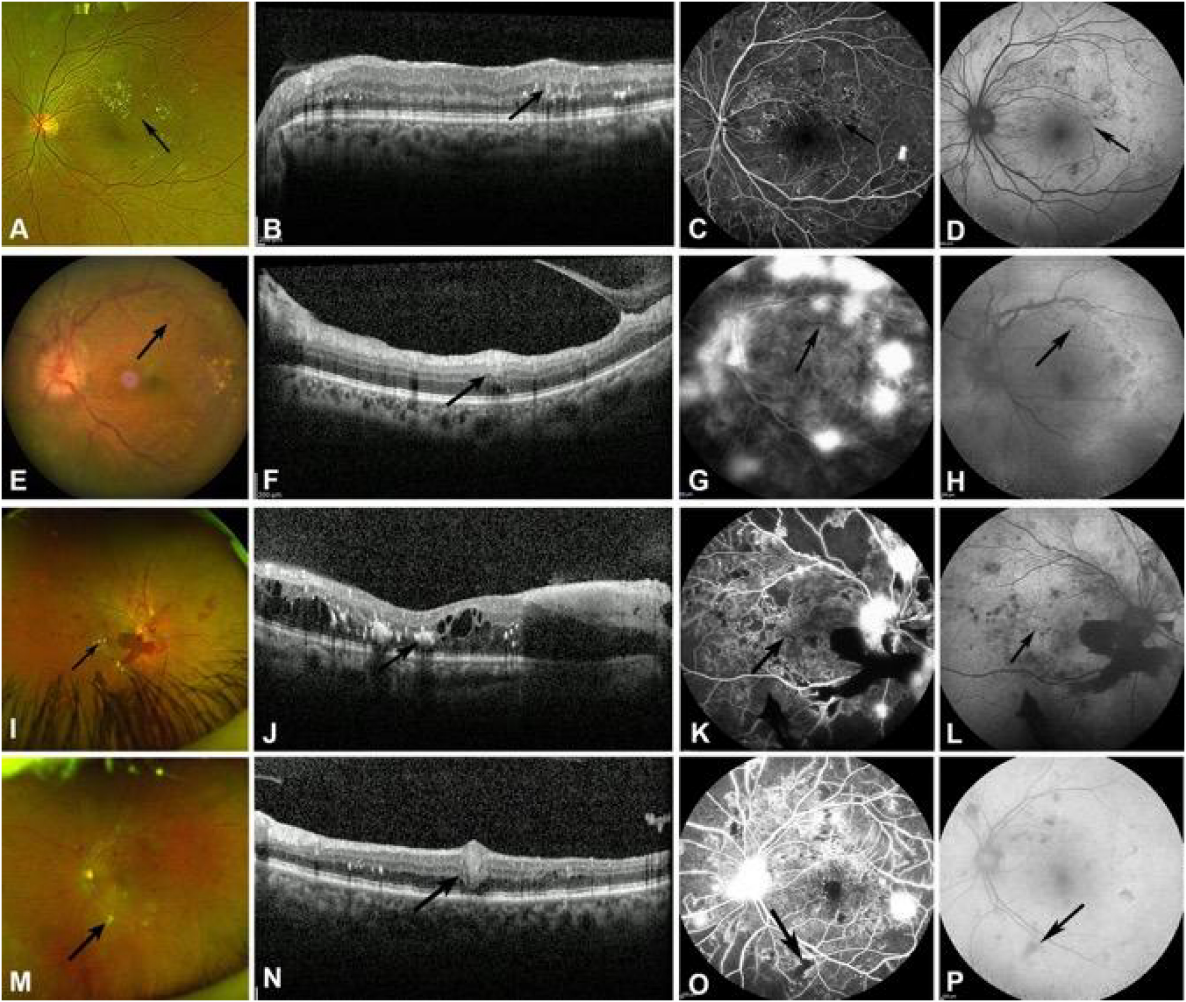
The manifestations of MA, intra-retinal hemorrhage, hard exudates, and cotton wool spots in PDR patients are observed in fundus photography, SD-OCT, FFA, and FAF examination. **(A)** Typical MA (black arrow) on fundus photography. **(B)** MA manifested as moderately reflective punctate lesions on SD-OCT (black arrow). **(C)** FFA shows microaneurysms as punctate hyperfluorescence at the same location (black arrow). **(D)** Weak fluorescence changes of MA on FAF (black arrow). **(E)** Intra-retinal hemorrhage (black arrow) on fundus photography. **(F)** Hyperreflective lesions with accompanying artifacts (black arrow) of intraretinal hemorrhage on SD-OCT. (G) Blocked fluorescence of retinal hemorrhage on FFA (black arrow). **(H)** Blocked fluorescence of the same hemorrhage on FAF (black arrow). **(I)** Hard exudate (black arrow) on fundus photography. **(J)** Hard exudate manifested as punctate hyperreflective materials in the outer plexiform layer of the retina, accompanied by posterior shadowing on SD-OCT (black arrow). **(K)** FFA shows a hard exudate as a hypofluorescent focus (black arrow). **(L)** Weak fluorescence changes of hard exudate on FAF (black arrow). **(M)** Cotton wool spot (black arrow) on fundus photography. **(N)** Thickened retinal nerve fiber layer lesions with high reflectivity (black arrow) of cotton wool spot on SD-OCT. **(O)** Hypofluorescence foci with NP areas underneath the cotton wool spot on FFA (black arrow). **(P)** Hypofluorescence foci of the same cotton wool spot on FAF (black arrow).

In PDR patients (Figure 3), fundus photography revealed cotton-wool spot changes in 48 of 99 eyes (48.5%), with 12 in the macula and the remaining 36 in the extra-macular retina. SD-OCT scans of the macula can reveal cotton-wool spots in patients with PDR. FFA showed hypofluorescent foci in 48 eyes of PDR patients, consistent with fundus photography findings. On FAF, 36.4% (36/99) of PDR eyes showed hypoautofluorescent cotton-wool spots, while the remaining 12 eyes showed no clear lesions. The transition from acute CWS to chronic DRIL in PDR patients reflects the progression of ischemic damage from the nerve fiber layer to the inner retinal layers.

#### Hyperreflective foci in the outer retina

**FIGURE 4:**
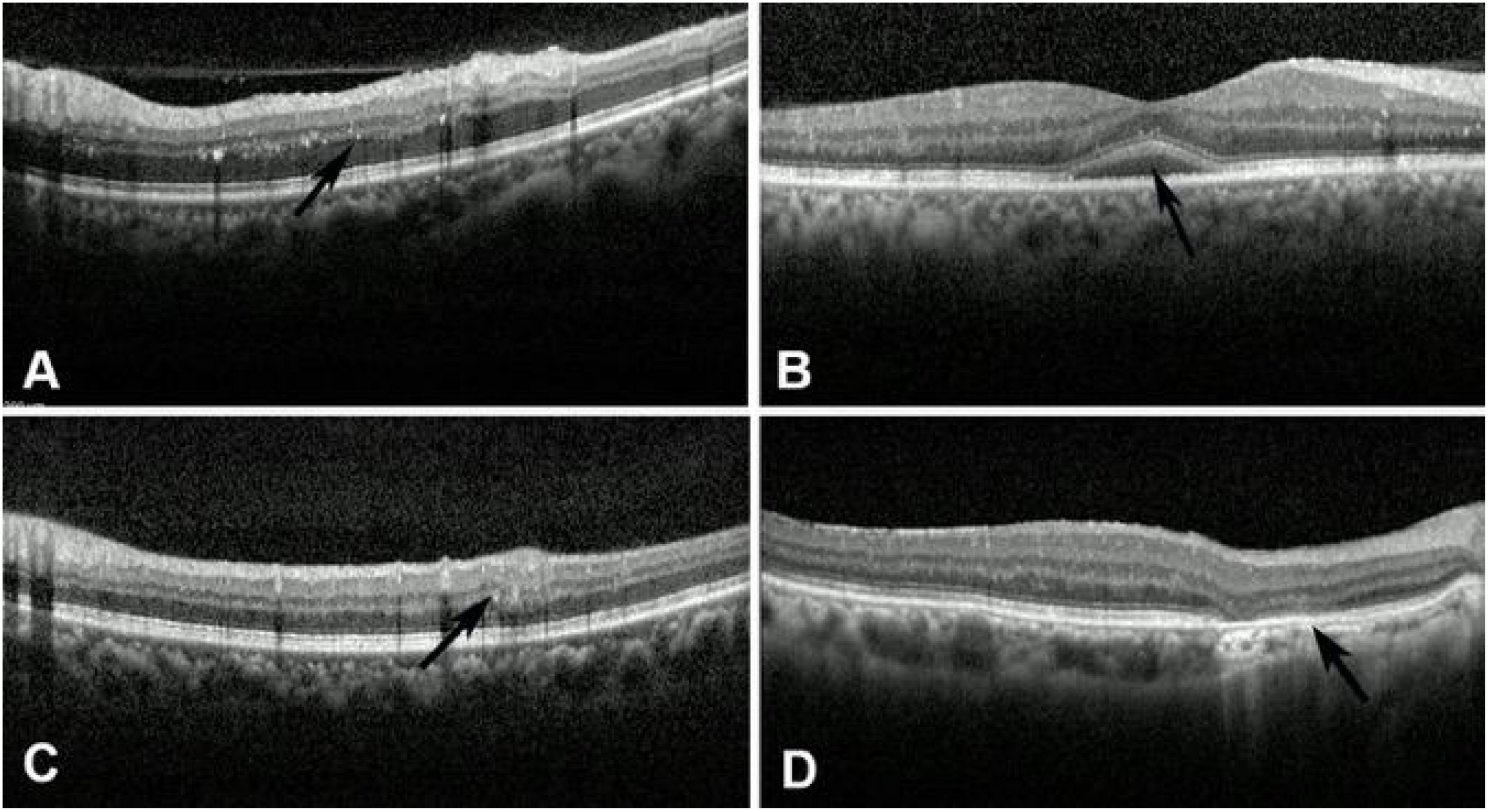
The imaging findings of the hyperreflective foci, SRF, DRLL, and disruption of ELM and EZ on SD-OCT. **(A)** Punctate medium-reflective substances with hyperreflective foci in the inner and outer retinal layers with no posterior shadowing (black arrow). **(B)** SRF was featured as macular serous neurosensory detachment with intact pigment epithelium (black arrow). **(C)** DRIL was manifested as irregular disorganization of the inner plexiform layer, inner nuclear layer, and outer plexiform layer (black arrow). **(D)** Disruption of ELM and EZ was featured as a reduction or loss of the ELM and EZ reflection signal (black arrow).

Spectral-domain optical coherence tomography revealed hyperreflective foci in 75 of 120 eyes (62.5%) in patients with NPDR. Among the 99 eyes of PDR patients, 87 eyes (87/99, 87.9%) exhibited hyperreflective foci. The hyperreflective foci on OCT appear as punctate, medium-reflective substances in the inner and outer retinal layers, with a diameter of less than 30 μm and no posterior shadowing (Figure 4A). It cannot be observed in the fundus photograph. Compared with hard exudates, the hyperreflective foci were smaller in size, and there was no posterior shadowing beneath the lesions. It was found that HRF is mostly located in the outer nuclear layer and outer plexiform layer of the retina, and their numbers increase as the condition worsens. Therefore, the frequency and number of HRFs are significantly higher in PDR than in NPDR. Additionally, HRF is not visible on fundus photography, FFA, or FAF examination.

#### Subretinal fluid and pigment epithelial detachment

Spectral-domain optical coherence tomography examination revealed that among 120 eyes of NPDR patients, SRF was observed in only 15 (12.5%). It was featured as a macular serous neurosensory detachment with intact pigment epithelium, showing a low-reflectance area formed by serious subretinal fluid between the neurosensory retina and the pigment epithelium (Figure 4B). In 99 eyes with PDR, SRF was found in 6 eyes (6/99, 6.1%). Our studies showed that SRF cannot be displayed in fundus photography, FFA, or FAF. Pigment epithelial detachment was not observed in any patient with NPDR or PDR on OCT in our study.

#### DRIL

In our study, SD-OCT revealed that among 120 patients with NPDR, DRIL was present in 27 eyes (22.5%, 27/120). It was manifested as irregular disorganization of the inner plexiform layer, inner nuclear layer, and outer plexiform layer in the lesion area, with loss of the normal retinal structure (Figure 4C). The formation of the DRLL may be closely related to macular ischemia. And in 99 eyes with PDR, DRIL was found in 27 eyes (27/99, 27.3%). Our study found that the location of DRIL essentially coincides with that of retinal cotton-wool spots; the presence of DRIL may indicate ischemic changes in the macular area of the retina.

#### Disruption of the ELM and the EZ

External limiting membrane and EZ are important retinal structures, as observed on OCT. SD-OCT revealed that among 120 eyes of NPDR patients, disruption of the ELM and EZ was observed in 18 eyes (15%; 18/120). ELM disruption on OCT is characterized by a reduction or loss of the ELM reflection signal, indicating structural or functional impairment of the ELM (Figure 4D). Disruption of EZ is characterized by a reduction or loss of the EZ reflective signal, indicating a decline in visual function. Compared with NPDR patients, SD-OCT revealed that, among 99 PDR eyes, disruption of the ELM and EZ was observed in 6 eyes (6/99, 6.1%). Our study shows that fundus photography, FFA, and FAF are all unable to visualize ELM and EZ disruption.

#### ERM

In our study, SD-OCT revealed epiretinal membranes in 80% (96/120) of eyes with NPDR, with varying degrees of severity. A typical epiretinal membrane on OCT appears as a hyperreflective band adherent to the inner retinal surface, either partially or completely attached. In some patients, the epiretinal membrane may contract, leading to retinal traction, which increases macular thickness and results in a diffuse reduction in the reflectivity of the inner retinal layers. In addition, some NPDR patients exhibit only mild epiretinal membranes, characterized by localized, small, hyperreflective bands on the retinal surface in the macula.

Compared to NPDR, 84.8% (84/99) of PDR patients exhibited varying degrees of ERM. The incidence of ERM in PDR patients was significantly higher than in NPDR patients, with the linear hyperreflective bands on the retinal surface being notably thicker in PDR patients. Retinal traction thickening was also more commonly observed in patients with PDR. FFA and FAF have difficulty observing ERM.

#### PVD

In our study, SD-OCT revealed that among 120 patients with NPDR, PVD was present in 54 eyes (45%). PVD can present as either partial or complete PVD. When complete PVD occurs, OCT reveals a medium-reflective band separated from the retina, appearing as an irregular curve or semicircular structure floating in the vitreous cavity. The interface between this medium-reflective band and the retina is clearly visible, and the retinal surface remains relatively smooth without retina folding. This feature can be used to differentiate it from ERM. When partial PVD occurs, the posterior vitreous cortex adheres to or exerts traction on the macular region. SD-OCT revealed a thin, medium-reflective line anterior to the macula, and may be accompanied by mild elevation of the fovea.

Compared with NPDR, SD-OCT revealed that among 99 PDR eyes, PVD was present in 72 (72/99, 72.7%). The incidence of PVD was significantly higher in PDR compared to NPDR. Additionally, in PDR patients with severe disease, the ERM is significantly thickened by traction, which should be differentiated from the medium-reflective band in PVD.

#### ICR and DME

**FIGURE 5:**
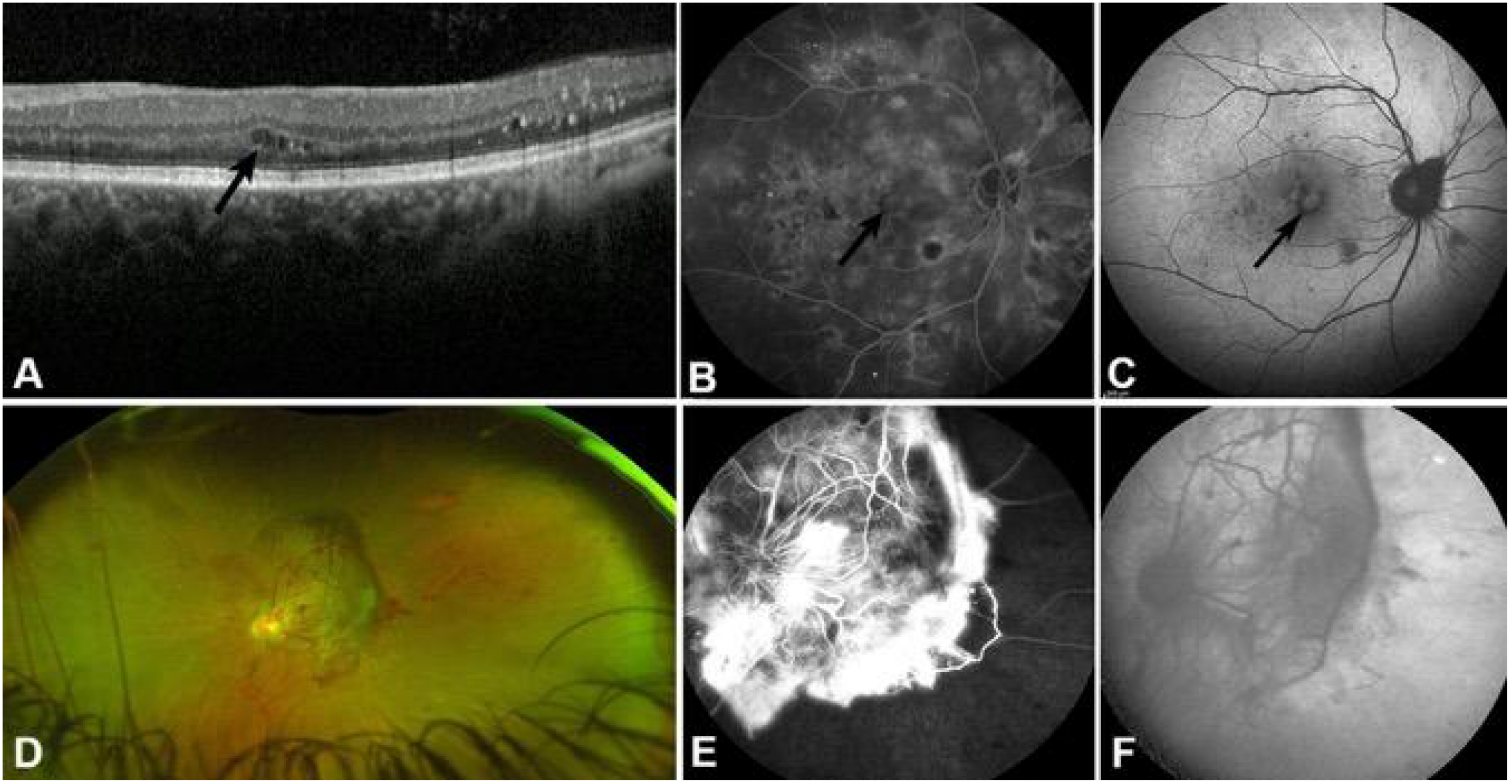
The imaging findings of the ICR and fibrous proliferative membrane on SD-OCT. **(A)** Small cystic cavities in the inner retinal layers of the macula (black arrow). **(B)** Cystoid macular edema in the late stage of FFA (black arrow). **(C)** FAF shows cystoid hyperfluorescence in the macula consistent with FFA (black arrow). **(D)** Neovascularization of the optic disk and fibrous proliferative membrane on fundus photography. **(E)** Leakage of neovascularization of the optic disk and staining of fibrous proliferative membrane on FFA. **(F)** Hypofluorescent fibroproliferative membrane on FAF.

In our study, SD-OCT revealed intraretinal cystic cavities in 39 of 120 eyes (32.5%) in patients with NPDR. It was characterized by one or more small cystic cavities in the inner retinal layers of the macula, primarily in the outer plexiform layer (Figure 5A). These cystic spaces appear as hyporeflective areas on OCT, forming a distinct contrast with normal retinal tissue. In addition, the presence of fluids in the cyst can lead to an increase in retinal thickness.

CME and DRT were two types of diabetic macular edema. CME is a manifestation of IRC, characterized by fluid accumulation in the intercellular spaces of the retinal neural layers within the macula, particularly in the outer plexiform layer (OPL) around the fovea. The loose Henle fibers in this area are especially prone to fluid accumulation. When the volume of fluid increases, it can push apart the fiber bundles, forming multiple cystic cavities, leading to CME. In NPDR patients, SD-OCT scans revealed macular edema in 48 eyes; 39 were accompanied by IRC, while the remaining 9 exhibited DRT. However, only six eyes exhibited cystoid macular edema (Figure 5B) in the late stage of FFA. Additionally, FAF revealed hyperfluorescent changes indicative of macular edema in 12 of 120 eyes (10%), with six eyes exhibiting typical cystoid macular hyperfluorescence (Figure 5C), consistent with FFA findings. Compared with NPDR patients, SD-OCT revealed IRC in 48 of 99 eyes (48.5%) and macular edema in 93 of 99 eyes (93.9%) in PDR patients, indicating a higher frequency of both IRC and macular edema in PDR. FAF revealed hyperfluorescence indicative of macular edema in 21 out of 99 eyes (21.2%), with 15 eyes (15.2%) exhibiting typical cystoid macular edema.

### The specific manifestations of PDR in SD-OCT, FAF, and FFA examination

#### Preretinal hemorrhage

Fundus photography revealed preretinal hemorrhage in 9 out of 99 eyes (9.1%) of PDR patients, manifesting as well-demarcated, large red lesions that may involve the macula. SD-OCT examination revealed preretinal hemorrhage in these nine eyes, manifesting as dense, hyperreflective material between the retinal nerve fiber layer and the posterior vitreous cortex, with significant artifacts obscuring the underlying tissue. On FFA, the hemorrhage appears as a blocking fluorescence, obscuring the underlying retinal vessels. On FAF, preretinal hemorrhage appears as hypofluorescence due to its ability to block RPE autofluorescence.

#### Fibrous proliferative membrane

Fundus photography shows yellowish-white membrane-like tissue on the surface of the optic disc or retina, with visible vascular patterns (Figure 5D). Spectral-domain optical coherence tomography examination revealed fibroproliferative changes in 27 out of 99 eyes (27.3%) of patients with PDR. Fibroproliferative membranes exhibit diverse OCT manifestations, typically appearing as hyperreflective bands adherent to the retinal surface, often accompanied by retinal structural disruption and thickening. Some patients exhibit distinct hyperreflective bands on the retinal surface, partially located within the vitreous cavity, with underlying retinal folds, inner retinal thickening, and disorganization. In severe cases of fibroproliferation, the retinal structure is completely disorganized, with the proliferative membrane appearing as an irregular hyperreflective band that obscures the underlying retinal tissue. Additionally, multiple irregularly shaped, variably sized cystic dark spaces can be observed between the proliferative membranes and the retina. In the FFA examination, severe cases of proliferative membrane exhibit a vascular network morphology with fluorescein leakage, accompanied by a large non-perfusion (NP) zone around the fibrotic membrane (Figure 5E). FAF revealed that 12 of 99 eyes (12.1%) in PDR patients showed a hypofluorescent fibroproliferative membrane, with partial visualization of vascular morphology in some cases (Figure 5F). This suggests that FAF demonstrates typical fibroproliferative membranes well.

#### SFCT

All patients underwent EDI-OCT examinations to measure the SFCT. The average SFCT was (244.72 ± 49.14) μm in the healthy control group, (287.73 ± 83.61) μm in the NPDR group, and (301.19 ± 82.44) μm in the PDR group. Compared with the healthy control group, the SFCT in the NPDR group was significantly thicker (*P* = 0.019 < 0.05). After adjusting for age using multivariate regression, the difference remained statistically significant (β = 0.24, *P* = 0.031 < 0.05). The SFCT of the PDR group was considerably thicker than that of the normal control group, with a statistically significant difference (*P* = 0.003 < 0.005). There was no statistically significant difference in SFCT between the NPDR and PDR groups.

## Discussion

DR is one of the microvascular complications caused by diabetes and a leading cause of blindness in adults (1). The treatment plan also varies with DR severity. FFA remains the gold standard for diagnosing and staging DR and plays a crucial role in treatment planning. However, FFA has limitations, and some patients are unable to undergo the procedure due to contraindications, such as allergies or abnormal liver and kidney function. FAF has been widely recognized for its advantages in diagnosing age-related macular degeneration (ARMD) (6), hereditary retinal diseases (7), and central serous chorioretinopathy (8). Retinal function as reflected by FAF in DR provides new insights into diagnosis and treatment planning. SD-OCT can detect subtle retinal structural changes, which are significant for evaluating DR progression and prognosis. By providing a side-by-side multimodal comparison, this work offers practical insights for clinical risk stratification, particularly identifying subclinical structural changes that traditional examinations might miss.

In our study, microaneurysms (MA) and intraretinal hemorrhages were observed in all DR patients, indicating that they are fundamental retinal lesions in DR. In OCT, MA appears as a medium-reflective, punctate lesion in the inner retinal layers, measuring greater than 30 μm in diameter, with weak fluorescence on FAF. Intraretinal hemorrhages appear as variably sized hyperreflective foci in the superficial and/or inner retinal layers on OCT, accompanied by blocking fluorescence in FAF. OCT examination revealed that MA primarily originates from the inner nuclear layer. A prospective study has demonstrated that a high MA formation rate is a biomarker for the development of clinically significant macular edema in patients with type 2 diabetes (9). Additionally, research has shown that MAs originating from the deep capillary layer are strongly correlated with CME (10). Compared with MA, intraretinal hemorrhages are more easily detected on FAF. Our comparison of FFA and OCT reveals that some small MAs may not be visible in FAF, possibly because the blocking fluorescence caused by intraretinal hemorrhages is more distinguishable against the background of retinal autofluorescence than the weak fluorescence produced by MAs. Similarly, FAF is highly effective for detecting preretinal hemorrhages. In our study, FAF detected preretinal hemorrhages in all PDR patients.

Hyperreflective foci was first described by Coscas et al. using OCT in age-related macular degeneration (11). In OCT, HRF appears as a medium-reflective focus with a diameter of less than 30 μm in the retina. The formation is primarily attributed to the aggregation of activated microglia within the retina, or the migration of monocytes from the blood into the retina, which differentiate into macrophages (12). These aggregates, due to their high metabolic activity or lipid components, manifest as HRF in OCT. Some studies suggest that HRF may also originate from extravasated lipoproteins, cellular debris, or metabolic byproducts (13). In our research, HRF was predominantly located in the inner retinal layers of both patients with NPDR and PDR. In our study, HRF was detected in 87 eyes of the PDR group and 75 eyes of the NPDR group. Additionally, the number of HRFs was significantly higher in the PDR group than in the NPDR group. HRF cannot be distinguished in fundus photography, FFA, or FAF. HRF has been observed in patients with wet AMD, CME, retinal vein occlusion (RVO), central serous chorioretinopathy (CSC), retinitis pigmentosa, and other conditions leading to choroidal neovascularization (14, 15). In recent years, HRF has attracted significant attention as a biomarker of DME treatment efficacy. The presence and increased number of HRFs indicate a poor response to anti-VEGF therapy in patients with DME.

Studies have shown a relationship between HRF and ELM disruption (16). Since the ELM prevents the infiltration of large molecules into the retina, it suggests that HRF may originate from damage to the blood-retinal barrier. The ELM provides structural support for glial cells and photoreceptors. Its instability can lead to misalignment of photoreceptors. The EZ, or ellipsoid zone, primarily consists of mitochondria. Damage to these structures can be directly visualized in OCT as ELM/EZ disruption or absence. Studies have shown that damage to ELM and EZ not only leads to the appearance or increase of HRF but also causes a significant decline in patients’ vision and a poorer prognosis (17). In this study, ELM and EZ disruption were observed in 18 eyes of the NPDR group and six eyes of the PDR group. However, the number of eyes with HRF was significantly higher than those with ELM or EZ disruption in both groups, indicating that HRF is associated not only with ELM disruption but also with other factors. In OCT, hard exudates appear as hyperreflective foci with a diameter greater than 30 μm, and some studies suggest that HRF may be a precursor to hard exudates. Mori et al. found that increased VEGF disrupts the integrity of the EZ and ELM (18). Therefore, observing changes in the EZ and ELM via OCT is of significant value for assessing retinal function and prognosis in patients with DR.

Disorganization of retinal inner layers refers to the irregular structural disruption of the inner plexiform layer (IPL), inner nuclear layer (INL), and outer nuclear layer (ONL), a common imaging feature in DR that may be associated with blood-retinal barrier breakdown and macular ischemia. Our study found that the structural disruption in the inner retinal layers at the cotton-wool spots on OCT is similar to that in DRLL. In OCT, cotton-wool spots appear as hyperreflective thickening of the nerve fiber layer with underlying artifacts. It has been reported that DRIL can reflect retinal capillary non-perfusion areas caused by ischemia (19). DRIL and cotton-wool spots are closely associated with retinal ischemia, potentially reflecting a cascade of ischemic damage from the nerve fiber layer to the inner retinal layers.

Diabetic macular edema can be classified into DRT and CME based on OCT examination results. In this study, it was found that DR patients can develop a mixed pattern of DME, which is consistent with the findings of Kim et al. (20). Soliman et al. found that large cysts in the outer nuclear layer and/or Henle layer on OCT correspond to petaloid cystic leakage on FFA (21). In our study, FFA revealed macular cystoid edema in 6 eyes (5%) of NPDR patients and 15 eyes (16.13%) of PDR patients, findings consistent with those of FAF. This suggests that FAF can serve as an alternative to FFA and OCT for observing typical macular cystoid edema, aligning with the results of McBain et al.

It has been shown that PVD is also involved in DME development (22). In diabetic patients, increased cross-linking of vitreous collagen and thickening of the posterior vitreous cortex may lead to macular traction and macular edema. PVD has both protective effects and is a risk factor for the worsening of DME. In DR patients with PVD, the incidence of macular edema is lower, and some patients experience a reduction in macular edema after PVD. On the other hand, when PVD is accompanied by retinal proliferation, it can exacerbate macular edema or lead to retinal tears or detachment. In our study, PVD was observed in 72 eyes of PDR patients, which was higher than in the 54 eyes of NPDR patients. Additionally, the incidence of DME was higher in PDR eyes than in NPDR eyes.

The formation of SRF is associated with increased vascular permeability, driven by mechanisms such as blood-retinal barrier disruption and elevated VEGF levels (23). In OCT, SRF appears as a hyporeflective area beneath the retinal neuroepithelium, with an intact RPE layer, suggesting that its formation results from cellular barrier dysfunction rather than structural damage. This finding is also supported by Urner-Bloch et al. (24). SRF can serve as a biological marker for evaluating DME and the therapeutic efficacy of anti-VEGF treatment. In our study, SRF was observed in 15 eyes of the NPDR group, compared to 6 eyes in the PDR group. The presence of SRF is associated with visual decline in patients. DME patients with SRF may exhibit better therapeutic efficacy or anatomical recovery after anti-VEGF treatment. Therefore, investigating SRF on OCT in DR patients can also serve as a metric for assessing the effectiveness of DME treatment.

In our study, we found that the proportion of ERM was very high in DR patients, both in eyes with NPDR and in those with PDR. This phenomenon has not been clinically recognized or emphasized. SD-OCT examination revealed ERM in 96 out of 120 eyes (80%) of NPDR patients and 84 out of 99 eyes (84.8%) of PDR patients, with varying degrees of severity. The high incidence of ERM suggests a close association with DR. This high prevalence is attributed to the inclusion of early-stage, subclinical ERMs, characterized by localized hyperreflective bands on SD-OCT, which are often underdetected by fundus photography but are clearly visible on high-definition line scans. The ERM in PDR eyes is more severe than that in NPDR eyes and may be accompanied by proliferation. The formation of ERM is closely associated with Müller cells, and fibroblast growth factor 2 (FGF2) has been shown to induce Müller cell activation in patients with PDR (25). In DR patients, the retina has already been damaged by diabetic microvascular disease. The traction of the ERM may exacerbate retinal ischemia and hypoxia, leading to changes in the inner retinal reflectivity and increased macular thickness, thereby accelerating the progression of DR. Additionally, studies have found inflammatory cells in the fibrovascular membranes of patients with PDR (26), which may be one of the factors contributing to the development of ERM. In our study, the fibrovascular membrane was well visualized in FAF, appearing as weak fluorescence, while the surrounding retinal and vascular structures exhibited abnormal tractional morphology. In contrast, FFA may show strong fluorescence from neovascularization, which can obscure the membrane’s morphology.

In our study, there was no statistically significant difference in SFCT between the NPDR and PDR groups. However, SFCT was significantly thicker in both NPDR and PDR patients compared with the healthy control group. The findings of Kim et al. (27) are consistent with ours, and Savage et al. (28) further found that choroidal blood flow was significantly reduced in patients with PDR after treatment. This suggests that the physiological state of the retina and the biochemical reactions occurring therein influence the normal function of the choroidal vasculature, leading to choroidal vasodilation, increased permeability, and, consequently, increased choroidal blood flow and thickness. A deeper exploration of the potential link between choroidal thickness and the pathogenesis of DR could provide a theoretical basis for DR treatment.

This study provides a comprehensive and multimodal characterization of imaging biomarkers in DR by integrating SD-OCT, fundus autofluorescence, and FAF. Unlike previous studies that focused on single imaging modalities or isolated biomarkers, our work systematically compared the detection capability of different imaging techniques across a broad spectrum of retinal lesions in both NPDR and PDR. Notably, we demonstrated a remarkably high prevalence of epiretinal membrane in DR, which is underrecognized in routine clinical evaluation and may be an important structural indicator of disease severity. In addition, this study highlights the complementary role of FAF in visualizing retinal hemorrhage, DME, and fibrovascular proliferative membranes, suggesting its potential value as a non-invasive adjunct when FFA is contraindicated. By providing a side-by-side comparison of multimodal imaging features, this work offers practical insights for clinical assessment and may contribute to improved phenotyping and risk stratification in DR.

This study has several limitations that should be acknowledged. First, this was a cross-sectional imaging study, which limits the ability to establish causal relationships or evaluate longitudinal changes of imaging biomarkers during disease progression. Second, although multiple OCT, FAF, and FFA features were systematically analyzed, quantitative measurements such as lesion area, volume, or intensity were not performed, and the analysis mainly relied on the presence or absence of imaging features. Third, OCT examinations were primarily focused on the macula, which may have led to underestimation of peripheral retinal lesions, particularly cotton-wool spots, hard exudates, and microaneurysms located outside the posterior pole. Fourth, patients with poor image quality due to media opacity, severe vitreous hemorrhage, or tractional retinal detachment were excluded, which may introduce selection bias and limit the generalizability of the findings to more advanced stages of diabetic retinopathy. Finally, potential systemic confounders, such as diabetes duration, glycemic control, and systemic treatment, were not incorporated into the imaging analysis and should be considered in future studies. Notably, the significant age gap between the control and DR groups is a major limitation. Although multivariate analysis was performed to adjust for age, a perfectly age-matched cohort would provide more robust results. Furthermore, the lack of AL measurements is a critical omission, as AL is a known determinant of choroidal thickness. Future studies should include AL as a covariate to fully validate the findings regarding choroidal thickening in DR. Regarding the results detected by ELM/EZ, The limited macular scan area may have led to an underestimation of total retinal structural disruption in PDR eyes, particularly in the peripheral retina.

## Conclusion

As a non-invasive retinal examination technique, FAF offers unique advantages for detecting retinal hemorrhage, cystoid macular edema, and retinal fibrovascular proliferative membranes. We have an important finding: SD-OCT revealed a significant increase in the incidence of macular epiretinal membranes in DR patients, with a higher prevalence in PDR than in DR. SD-OCT can detect multiple imaging biomarkers in DR patients. By integrating features from FAF, fundus photography, and FFA, this comprehensive analysis aids in diagnosing DR and provides effective prognostic predictions for DR patients.

## Data Availability

The raw data supporting the conclusions of this article will be made available by the authors, without undue reservation.

## References

[1] Pushparani D Varalakshmi J Roobini K Hamshapriya P Livitha A. Diabetic retinopathy-A review. *Curr Diabetes Rev.* (2025) 21:43–55. 10.2174/0115733998296228240521151050 38831577

[2] Gbd 2019 Blindness and Vision Impairment Collaborators, Vision Loss Expert Group of the Global Burden of Disease Study. Causes of blindness and vision impairment in 2020 and trends over 30 years, and prevalence of avoidable blindness in relation to VISION 2020: the right to Sight: an analysis for the Global burden of disease study. *Lancet Glob Health.* (2021) 9:e144–60. 10.1016/S2214-109X(20)30489-7 33275949 PMC7820391

[3] Tan H Wang X Ye K Lin J Song E Gong L. Prevalence and risk factors of diabetic retinopathy among Chinese adults with type 2 diabetes in a suburb of Shanghai, China. *PLoS One.* (2022) 17:e0275617. 10.1371/journal.pone.0275617 36194621 PMC9531829

[4] Perais J Agarwal R Evans J Loveman E Colquitt J Owens D Prognostic factors for the development and progression of proliferative diabetic retinopathy in people with diabetic retinopathy. *Cochrane Database Syst Rev.* (2023) 2:CD013775. 10.1002/14651858.CD013775.pub2 36815723 PMC9943918

[5] Hee M Puliafito C Duker J Reichel E Coker J Wilkins J Topography of diabetic macular edema with optical coherence tomography. *Ophthalmology.* (1998) 105:360–70. 10.1016/s0161-6420(98)93601-6 9479300 PMC2923575

[6] Bindewald A Bird A Dandekar S Dolar-Szczasny J Dreyhaupt J Fitzke F Classification of fundus autofluorescence patterns in early age-related macular disease. *Invest Ophthalmol Vis Sci.* (2005) 46:3309–14. 10.1167/iovs.04-0430 16123434

[7] Pichi F Abboud E Ghazi N Khan A. Fundus autofluorescence imaging in hereditary retinal diseases. *Acta Ophthalmol.* (2018) 96:e549–61. 10.1111/aos.13602 29098804

[8] Han J Cho N Kim K Kim E Kim D Kim J Fundus autofluorescence patterns in central serous chorioretinopathy. *Retina.* (2020) 40:1387–94. 10.1097/IAE.0000000000002580 31157711 PMC7302330

[9] Nunes S Pires I Rosa A Duarte L Bernardes R Cunha-Vaz J. Microaneurysm turnover is a biomarker for diabetic retinopathy progression to clinically significant macular edema: findings for type 2 diabetics with nonproliferative retinopathy. *Ophthalmologica.* (2009) 223:292–7. 10.1159/000213639 19372723

[10] Hasegawa N Nozaki M Takase N Yoshida M Ogura Y. New insights into microaneurysms in the deep capillary plexus detected by optical coherence tomography angiography in diabetic macular edema. *Invest Ophthalmol Vis Sci.* (2016) 57:OCT348–55. 10.1167/iovs.15-18782 27409492

[11] Vidal-Oliver L Montolío-Marzo E Gallego-Pinazo R Dolz-Marco R. Optical coherence tomography biomarkers in early and intermediate age-related macular degeneration: a clinical guide. *Clin Exp Ophthalmol.* (2024) 52:207–19. 10.1111/ceo.14337 38214056

[12] Zeng H Green W Tso M. Microglial activation in human diabetic retinopathy. *Arch Ophthalmol.* (2008) 126:227–32. 10.1001/archophthalmol.2007.65 18268214

[13] Bolz M Schmidt-Erfurth U Deak G Mylonas G Kriechbaum K Scholda C. Diabetic retinopathy research group Vienna. Optical coherence tomographic hyperreflective foci: a morphologic sign of lipid extravasation in diabetic macular edema. *Ophthalmology.* (2009) 116:914–20. 10.1016/j.ophtha.2008.12.039 19410950

[14] Arthi M Sindal M Rashmita R. Hyperreflective foci as biomarkers for inflammation in diabetic macular edema: retrospective analysis of treatment naïve eyes from south India. *Indian J Ophthalmol.* (2021) 69:1197–202. 10.4103/ijo.IJO_2627_20 33913858 PMC8186614

[15] Wang Z Zhou J Hu X Ding J Zhang X Wei R Exploring potential imaging biomarkers for ischemic retinal vein occlusion: insights from fundus autofluorescenceand optical coherence tomography. *Front. Med.* (2025) 12:1726962. 10.3389/fmed.2025.1726962 41480538 PMC12755151

[16] Uji A Murakami T Nishijima K Akagi T Horii T Arakawa N Association between hyperreflective foci in the outer retina, status of photoreceptor layer, and visual acuity in diabetic macular edema. *Am J Ophthalmol.* (2012) 153:710–7.e1. 10.1016/j.ajo.2011.08.041 22137207

[17] Maheshwary A Oster S Yuson R Cheng L Mojana F Freeman W. The association between percent disruption of the photoreceptor inner segment-outer segment junction and visual acuity in diabetic macular edema. *Am J Ophthalmol.* (2010) 150:63–7.e1. 10.1016/j.ajo.2010.01.039 20451897 PMC2900476

[18] Mori Y Suzuma K Uji A Ishihara K Yoshitake S Fujimoto M Restoration of foveal photoreceptors after intravitreal ranibizumab injections for diabetic macular edema. *Sci Rep.* (2016) 6:39161. 10.1038/srep39161 27966644 PMC5155247

[19] McLeod D. Why cotton wool spots should not be regarded as retinal nerve fibre layer infarcts. *Br J Ophthalmol.* (2005) 89:229–37. 10.1136/bjo.2004.058347 15665358 PMC1772507

[20] Kim B Smith S Kaiser P. Optical coherence tomographic patterns of diabetic macular edema. *Am J Ophthalmol.* (2006) 142:405–12. 10.1016/j.ajo.2006.04.023 16935584

[21] Soliman W Sander B Hasler P Larsen M. Correlation between intraretinal changes in diabetic macular oedema seen in fluorescein angiography and optical coherence tomography. *Acta Ophthalmol.* (2008) 86:34–9. 10.1111/j.1600-0420.2007.00989.x 17651471

[22] Yamaguchi Y Otani T Kishi S. Resolution of diabetic cystoid macular edema associated with spontaneous vitreofoveal separation. *Am J Ophthalmol.* (2003) 135:116–8. 10.1016/s0002-9394(02)01855-x 12504718

[23] Apte R Chen D Ferrara N. VEGF in signaling and disease: beyond discovery and development. *Cell.* (2019) 176:1248–64. 10.1016/j.cell.2019.01.021 30849371 PMC6410740

[24] Urner-Bloch U Urner M Jaberg-Bentele N Frauchiger A Dummer R Goldinger SM. MEK inhibitor-associated retinopathy (MEKAR) in metastatic melanoma: long-term ophthalmic effects. *Eur J Cancer.* (2016) 65:130–8. 10.1016/j.ejca.2016.06.018 27497344

[25] Rezzola S Guerra J Krishna Chandran A Loda A Cancarini A Sacristani P VEGF-independent activation of müller cells by the vitreous from proliferative diabetic retinopathy patients. *Int J Mol Sci.* (2021) 22:2179. 10.3390/ijms22042179 33671690 PMC7926720

[26] Urbančič M Štunf Š Milutinović Živin A Petrovič D GlobočnikPetrovič M. Epiretinal membrane inflammatory cell density might reflect the activity of proliferative diabetic retinopathy. *Invest Ophthalmol Vis Sci.* (2014) 55:8576–82. 10.1167/iovs.13-13634 25425310

[27] Kim J Lee D Joe S Kim J Yoon Y. Changes in choroidal thickness in relation to the severity of retinopathy and macular edema in type 2 diabetic patients. *Invest Ophthalmol Vis Sci.* (2013) 54:3378–84. 10.1167/iovs.12-11503 23611988

[28] Savage H Hendrix J Peterson D Young H Wilkinson C. Differences in pulsatile ocular blood flow among three classifications of diabetic retinopathy. *Invest Ophthalmol Vis Sci.* (2004) 45:4504–9. 10.1167/iovs.04-0077 15557461

